# The Multifaceted Role of Nutrient Sensing and mTORC1 Signaling in Physiology and Aging

**DOI:** 10.3389/fragi.2021.707372

**Published:** 2021-08-27

**Authors:** Stephanie A. Fernandes, Constantinos Demetriades

**Affiliations:** ^1^ Max Planck Institute for Biology of Ageing (MPI-AGE), Cologne, Germany; ^2^ Cologne Graduate School for Ageing Research (CGA), Cologne, Germany; ^3^ University of Cologne, Cologne Excellence Cluster on Cellular Stress Responses in Aging-Associated Diseases (CECAD), Cologne, Germany

**Keywords:** nutrient sensing, mTORC1, aging, dietary restriction, amino acids

## Abstract

The mechanistic Target of Rapamycin (mTOR) is a growth-related kinase that, in the context of the mTOR complex 1 (mTORC1), touches upon most fundamental cellular processes. Consequently, its activity is a critical determinant for cellular and organismal physiology, while its dysregulation is commonly linked to human aging and age-related disease. Presumably the most important stimulus that regulates mTORC1 activity is nutrient sufficiency, whereby amino acids play a predominant role. In fact, mTORC1 functions as a molecular sensor for amino acids, linking the cellular demand to the nutritional supply. Notably, dietary restriction (DR), a nutritional regimen that has been shown to extend lifespan and improve healthspan in a broad spectrum of organisms, works via limiting nutrient uptake and changes in mTORC1 activity. Furthermore, pharmacological inhibition of mTORC1, using rapamycin or its analogs (rapalogs), can mimic the pro-longevity effects of DR. Conversely, nutritional amino acid overload has been tightly linked to aging and diseases, such as cancer, type 2 diabetes and obesity. Similar effects can also be recapitulated by mutations in upstream mTORC1 regulators, thus establishing a tight connection between mTORC1 signaling and aging. Although the role of growth factor signaling upstream of mTORC1 in aging has been investigated extensively, the involvement of signaling components participating in the nutrient sensing branch is less well understood. In this review, we provide a comprehensive overview of the molecular and cellular mechanisms that signal nutrient availability to mTORC1, and summarize the role that nutrients, nutrient sensors, and other components of the nutrient sensing machinery play in cellular and organismal aging.

## Introduction

Aging is characterized by a progressive decline in multiple cellular and organismal functions, collectively described as “the hallmarks of aging” (reviewed in Lopez-Otin et al. (2013)). A fundamental process that—directly or indirectly—influences virtually all other pillars of aging is the ability of cells to sense the presence or absence of intra- and extra-cellular nutrients. Accordingly, dysregulation of the cellular nutrient sensing machinery is tightly linked to cellular dysfunction in aged tissues. At the molecular level, a serine/threonine protein kinase called mechanistic Target of Rapamycin (mTOR) functions both as the main nutrient sensor and a key controller of cellular metabolism. mTOR participates in two distinct multiprotein complexes, namely mTOR complex 1 (mTORC1) and mTOR complex 2 (mTORC2), which differ in their composition, upstream regulation, and downstream functions. Although both complexes respond to growth factor signaling, nutrients—like amino acids and glucose—signal mainly via mTORC1. When nutrients are abundant, active mTORC1 promotes anabolic processes, such as protein, nucleotide and lipid biosynthesis, while inhibiting processes mediating protein and organelle turnover, like autophagy and lysosomal biogenesis (Kim and Guan, 2019). Consistently, mTORC1 is readily inactivated upon nutrient starvation, or in response to a multitude of cellular stresses, to cease growth. As a central node in sensing environmental cues and translating them to cellular growth and metabolism, it is not surprising that mTORC1 activity is commonly dysregulated upon aging and in most age-related maladies, including cancer, metabolic diseases, and neurological disorders (Liu and Sabatini, 2020). Because, in some cases, mTORC1 activity is aberrantly elevated in aged cells (Chen et al., 2009), pharmacological or nutritional ways to target and inhibit this complex have been the focus of intense research over the last several decades.

In line with the well-established role of mTORC1 in nutrient sensing and in aging, dietary restriction, a nutritional regimen that positively influences lifespan and healthspan in a large panel of model organisms, including disease models, functions primarily via downregulating mTORC1. Limitation of protein intake, in particular, has been shown to lower mTORC1 activity in tumors and somatic tissues (Lamming et al., 2015), and to have beneficial effects on various health-related parameters, including insulin resistance, type 2 diabetes, obesity and mortality (Lagiou et al., 2007; Sluijs et al., 2010; Lagiou et al., 2012; Vergnaud et al., 2013; Levine et al., 2014; Fontana et al., 2016). This growing body of evidence highlights amino acids as key nutrients that are relevant for aging and disease, and amino acid sensing as an important cellular function that determines physiological and pathological mTORC1 signaling. Here, we comprehensively summarize the cellular mechanisms and principles of mTORC1 regulation by upstream stimuli, focusing primarily on the intricate network that signals amino acid availability to control mTORC1 activity and thus cellular growth and metabolism. Additionally, we discuss how nutritional, genetic and pharmacological perturbations that influence mTORC1 activity affect aging and age-associated diseases, and describe the role of upstream mTORC1 regulators, and downstream cellular functions in these processes.

## The Physiological Role of MTORC1 in Growth and Metabolism

### Composition of mTORC1

mTOR is a highly conserved serine/threonine protein kinase that belongs to the PIKK (PI3K-related protein kinases) family (Richardson et al., 2004b). In mammals, it exists as the catalytic component of two complexes, mTORC1 and mTORC2, which display distinct qualities (Liu and Sabatini, 2020). In this review, we focus primarily on mTORC1, which functions as the master nutrient sensor in cells. A characteristic subunit of mTORC1, with both functional and structural roles, is RAPTOR (regulatory-associated protein of mTOR) (Hara et al., 2002; Kim et al., 2002). It acts as a scaffold protein that is necessary for the integrity of the complex, and facilitates substrate recognition via binding sequences in certain mTORC1 targets, called TOS (TOR signaling motifs) (Nojima et al., 2003; Schaim et al., 2003). A second mTORC1 component is mLST8 (mammalian lethal with SEC13 protein 8; also known as GβL) (Kim et al., 2003). Although mLST8 was shown to act by enhancing the interaction between RAPTOR and mTOR, other reports suggested that mTORC1 activity is not affected in its absence (Guertin et al., 2006; Yang et al., 2013). Two additional mTORC1 subunits, PRAS40 (proline-rich AKT substrate 40 kDa; also known as AKT1S1) (Sancak et al., 2007; Vander Haar et al., 2007) and DEPTOR (DEP-domain-containing mTOR-interacting protein) (Peterson et al., 2009) interact with the complex via binding to RAPTOR and mTOR, respectively, and were shown to act as endogenous inhibitors of mTORC1 kinase activity.

### Role of mTORC1 in the Regulation of Protein Synthesis

**FIGURE 1:**
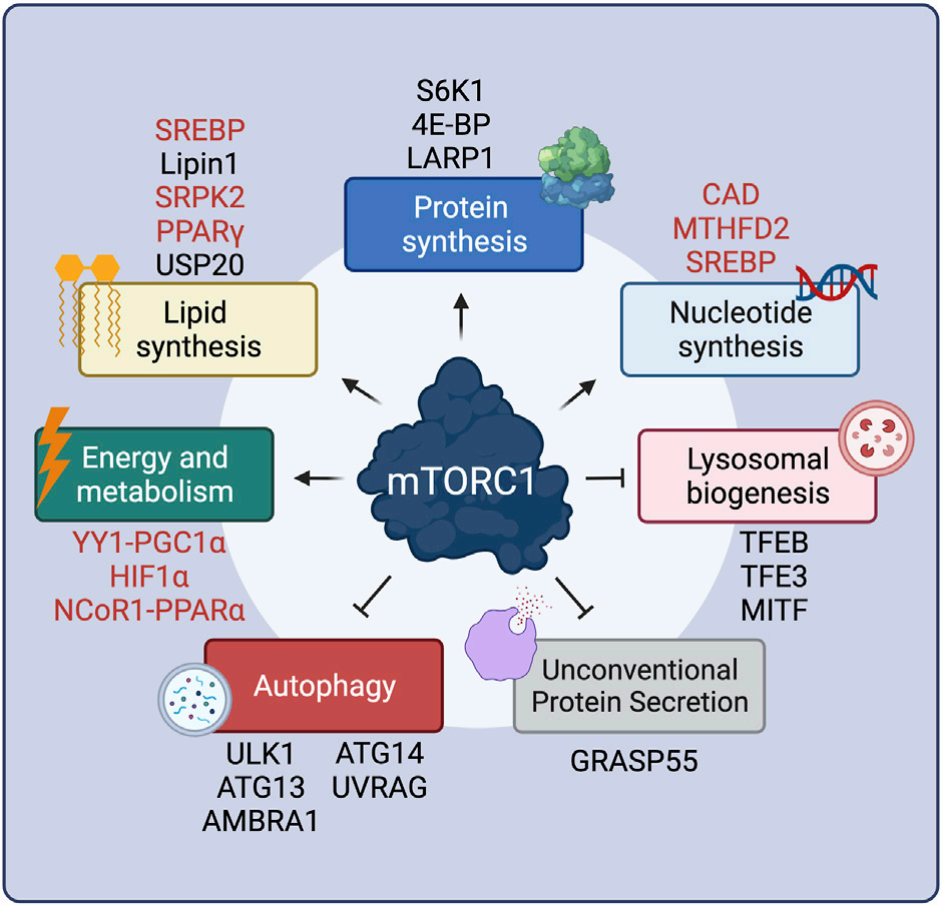
mTORC1 positively or negatively regulates multiple cellular functions via key effector proteins. Proteins that are regulated by mTORC1 only indirectly are shown in red. See text for details. Created with BioRender.com

Presumably the best-characterized downstream mTORC1 process is protein synthesis, a highly energy-consuming cellular function. By mTORC1 acting simultaneously as a central sensor for nutrient availability and the overall cellular metabolic status, and as a regulator of protein synthesis, cells ensure that they only make proteins when they have sufficient building blocks and energy. mTORC1 controls protein synthesis in many different ways, the best-studied being via phosphorylation of its canonical substrates, 4E-BP (eukaryotic initiation factor 4E binding proteins) and S6K1 (p70 S6 Kinase 1) proteins, both of which contain TOS motifs (Burnett et al., 1998; Nojima et al., 2003; Schaim et al., 2003). 4E-BP1 functions as a negative regulator of 5′-cap-dependent translation, via binding to eIF4E (eukaryotic translation initiation factor 4E). Multisite phosphorylation of 4E-BP1 by mTORC1 reduces its inhibitory interaction with eIF4E, thus promoting translation (Hara et al., 1997; Gingras et al., 1999) (Figure 1). A recent study uncovered the structural basis of 4E-BP1 phosphorylation by mTORC1, showing that 4E-BP1 is tethered to mTORC1 by two distinct interactions, which explain the hierarchical mode of 4E-BP1 phosphorylation (Bohm et al., 2021). In addition, 4E-BP1 can be phosphorylated by mTORC1 both in its free state and in the eIF4E-bound form, to guarantee that all pools of 4E-BP1 are targeted and ensure efficient translation initiation when mTORC1 is active (Bohm et al., 2021). mTORC1 is also known to favor translation of TOP (terminal oligopyrimidine tract) mRNAs, which bear a 5′ oligopyrimidine sequence downstream of the N^7^-methyl guanosine triphosphate cap, present in most ribosomal mRNAs (Levy et al., 1991; Hsieh et al., 2012; Thoreen et al., 2012). Although previous studies implied 4E-BP1 as the key factor in TOP mRNA translation (Hsieh et al., 2012; Thoreen et al., 2012), a recent report showed that mTORC1 directly phosphorylates and inactivates LARP1 (La-related protein 1), another important repressor of TOP mRNA translation (Jia et al., 2021) (Figure 1).

Unlike its inhibitory effect on 4E-BP1, mTORC1-driven S6K1 phosphorylation (Burnett et al., 1998) actually leads to its activation, and to subsequent phosphorylation of its downstream substrates (Figure 1). Full S6K1 activation also requires its phosphorylation by a second factor, PDK1 (Phosphoinositide-dependent kinase-1), which takes place at distinct residues (Alessi et al., 1998; Pullen et al., 1998). The best-described S6K1 target is S6 (ribosomal protein S6), although the function of its phosphorylation is still unclear, with some studies reporting that S6 phosphorylation is required for translation of ribosomal genes (Chauvin et al., 2014), whereas others showing that non-phosphorylatable S6 mutants have no effects on translation (Ruvinsky et al., 2005). S6K1 also activates transcription of ribosomal RNAs by enhancing RNA polymerase I activity via directly phosphorylating the regulatory factors UBF (upstream binding factor) (Hannan et al., 2003) and TIF-1A (transcription initiation factor 1A) (Mayer et al., 2004). Similarly, phosphorylation of the RNA polymerase III inhibitor MAF1 (repressor of RNA polymerase III transcription MAF1 homolog) (Shor et al., 2010) was also shown to be controlled by S6K1, although a second report suggested that MAF1 is a direct target of mTORC1 (Michels et al., 2010). In the context of protein translation initiation, S6K1 phosphorylates and activates eIF4B (eukaryotic translation initiation factor 4B), a positive regulator of the 5′-cap-binding eIF4F (eukaryotic translation initiation factor 4F) complex, thus promoting translation of mRNAs with complex 5′ untranslated regions (Holz et al., 2005). Additional S6K1 targets that are involved in translation are the negative regulator of the RNA helicase eIF4A (eukaryotic translation initiation factor 4A), PDCD4 (programmed cell death protein 4) (Dorrello et al., 2006), as well as the exon junction complex subunit SKAR (also known as POLDIP3; polymerase delta-interacting protein 3) that, when phosphorylated by S6K1, increases the efficiency of translation of spliced mRNAs (Richardson et al., 2004a; Ma et al., 2008).

### Role of mTORC1 in the Regulation of Lipid Biosynthesis

Cell growth requires concurrent membrane biogenesis, hence, mTORC1 is logically also involved in the regulation of lipid synthesis via various mechanisms (Figure 1). First, via S6K1, mTORC1 activates the SREBP (sterol regulatory element-binding proteins) transcription factors to promote *de novo* lipid biosynthesis (Porstmann et al., 2008; Duvel et al., 2010). Furthermore, mTORC1 controls expression of lipid biosynthesis genes by directly phosphorylating Lipin1, a negative regulator of SREBP, thus preventing its nuclear entry and allowing SREBP-dependent gene expression (Peterson et al., 2011). The mTORC1-S6K1 axis also promotes phosphorylation of SRPK2 (SRSF protein kinase 2), and, in concert with its phosphorylation by CK1 (casein kinase 1), induces SRPK2 nuclear translocation and the splicing of lipogenic-related transcripts (Lee et al., 2017). Additionally, the activity of PPARγ (peroxisome proliferator–activated receptor-γ), a transcription factor that promotes adipogenesis, is also tightly regulated by mTORC1 (Kim and Chen, 2004). Recently, a new layer in the regulation of lipid synthesis by mTORC1 was uncovered, with the finding that the levels of HMGCR (3-hydroxy-3-methylglutaryl-coenzyme A reductase), a rate-limiting enzyme in cholesterol biosynthesis are controlled by the activity of the deubiquitinase enzyme USP20, a direct mTORC1 substrate (Lu X.-Y. et al., 2020).

### Role of mTORC1 in the Regulation of Nucleotide Biosynthesis

As for the aforementioned processes, cell growth and proliferation also require increased DNA replication and rRNA (ribosomal RNA) production (Figure 1). In this regard, pyrimidine biosynthesis is also controlled downstream of mTORC1, via S6K1-mediated phosphorylation of CAD (carbamoyl-phosphate synthetase 2, aspartate transcarbamoylase, dihydroorotase), the rate-limiting enzyme catalyzing the first three steps in the pyrimidine biosynthesis pathway (Ben-Sahra et al., 2013; Robitaille et al., 2013). Similarly, mTORC1 also regulates purine biosynthesis by regulating the levels of MTHFD2 (mitochondrial tetrahydrofolate cycle enzyme methylenetetrahydrofolate dehydrogenase 2) via the transcription factor ATF4 (cyclic AMP-dependent transcription factor ATF-4) (Ben-Sahra et al., 2016). Finally, mTORC1 upregulates the oxidative arm of the pentose phosphate pathway, via SREBP, as an additional way of fueling nucleotide biosynthesis (Duvel et al., 2010).

### Role of mTORC1 in Energy Production and Metabolism

Because all cellular biosynthetic activities are energy-consuming processes, mTORC1 also controls energy production and metabolism (Figure 1). Via 4E-BP1, mTORC1 induces the expression of nuclear-encoded mitochondrial transcripts to boost ATP production (Morita et al., 2013). Moreover, mTORC1 also regulates mitochondrial biogenesis via the transcriptional YY1 (yin–yang 1) − PGC1α (PPARγ coactivator 1α) complex (Cunningham et al., 2007; Blattler et al., 2012). In parallel to promoting increased mitochondrial energy production, mTORC1 also acts on metabolic pathways that support cell growth, either by providing energy in the form of ATP (adenosine triphosphate) or by supplying precursors required for macromolecule biosynthesis. The best-studied example of mTORC1-mediated metabolic regulation is its role in the regulation of HIF1α (hypoxia inducible factor 1α) levels both via transcriptional (Duvel et al., 2010; He et al., 2018) and translational mechanisms (Zhong et al., 2000; Laughner et al., 2001; Hudson et al., 2002; Zid et al., 2009). Increased HIF1α, in turn, promotes several anabolic cellular functions, including glycolysis, a main energy-producing metabolic pathway (Duvel et al., 2010; He et al., 2018). In the liver, mTORC1 suppresses feeding- and aging-induced ketogenesis, presumably through promoting the nuclear relocalization of NCoR1 (nuclear receptor corepressor 1) to inhibit PPARα (peroxisome proliferator-activated receptor α)-mediated expression of ketogenic genes, thus coordinating hepatic ketone body production and the response to fasting/feeding (Sengupta et al., 2010; Kim K. et al., 2012).

### Role of mTORC1 in Autophagy Inhibition, Lysosomal Biogenesis and Unconventional Protein Secretion

Concomitantly with the upregulation of anabolic processes, mTORC1 also inhibits catabolic cellular functions, thus preventing degradation of cellular components and macromolecules that are necessary for cell growth (Figure 1). One such process is autophagy, via which cytoplasmic parts, damaged proteins and organelles are directed to lysosomes for degradation, facilitating the recycling of cellular components. Autophagy is a complex, multi-step process, with mTORC1 controlling several stages via the phosphorylation of key components of the autophagic machinery. When mTORC1 is inactive, autophagy induction is coordinated by an initiation complex formed by FIP200 (200-kDa FAK family kinase-interacting protein), ATG101 (autophagy-related protein 101), ULK1 (serine/threonine-protein kinase ULK1), and ATG13 (autophagy-related protein 13). Active mTORC1 phosphorylates and inactivates ULK1 (Kim et al., 2011) and ATG13 (Ganley et al., 2009; Hosokawa et al., 2009), thereby inhibiting autophagy initiation. Another target of mTORC1 involved in autophagy initiation is AMBRA1 (activating molecule in BECN1-regulated autophagy protein 1), which is crucial for ULK1 protein stability (Nazio et al., 2013). Moreover, mTORC1 inhibits the activity of the PIK3C3 (phosphatidylinositol 3-kinase catalytic subunit type 3) protein complex (also known as VPS34 complex) that is involved in the early steps of autophagy, via directly phosphorylating the ATG14 (autophagy-related protein 14) component (Yuan et al., 2013). Similarly, mTORC1 phosphorylates UVRAG (UV radiation resistance-associated gene product), a component involved in autophagosome maturation, thus inhibiting this process (Kim et al., 2015).

In addition to the control of autophagy by directly regulating components of the autophagic machinery, mTORC1 also controls the expression of genes involved in autophagy and lysosomal biogenesis by directly phosphorylating TFEB (transcription factor EB), as well as the related factors MITF (microphthalmia-associated transcription factor) and TFE3 (transcription factor E3) (Settembre et al., 2012). By phosphorylating TFEB, mTORC1 promotes its interaction with 14-3-3 proteins, which causes its cytoplasmic retention (Martina et al., 2012; Roczniak-Ferguson et al., 2012; Martina and Puertollano, 2013). In contrast, when mTORC1 is inactive, calcineurin (serine/threonine-protein phosphatase 2B) acts by dephosphorylating TFEB, thus promoting its nuclear translocation (Medina et al., 2015). Nuclear TFEB then promotes a transcriptional program which induces the expression of autophagy-related genes like UVRAG, as well as core lysosomal genes, such as vacuolar (H^+^)-ATPase (v-ATPase) subunits (Sardiello et al., 2009; Palmieri et al., 2011). Finally, a recent study showed that mTORC1 can drive the specific degradation of mRNAs coding for autophagy-related genes via stabilizing the m^6^A methyltransferase complex (MCT) (Tang et al., 2021). In sum, the tight connection between mTORC1 and the cellular recycling machinery ensures that cells follow an anabolic program while inhibiting catabolism when conditions are optimal. Accordingly, because autophagy activation causes the release of nutrients via the degradation of complex macromolecules, it also mediates the reactivation of mTORC1 to prevent excessive self-eating and maintain a fine balance between anabolism and catabolism in cells (Yu et al., 2010).

The only other cellular function that is inhibited by active mTORC1—besides autophagy and lysosomal biogenesis—is unconventional protein secretion (UPS), a process through which cells reshape their extracellular proteome in response to stress. The Golgi-residing GRASP55 protein is a key player in cargo transport via UPS routes. In unchallenged cells, mTORC1 phosphorylates directly GRASP55 at the Golgi to retain its subcellular localization. In contrast, inactivation of mTORC1 upon starvation or other cellular stresses leads to GRASP55 dephosphorylation and relocalization to secretory compartments of UPS, such as autophagosomes and multivesicular bodies (MVBs), to promote UPS of selected cargoes (Nüchel et al., 2021). Therefore, the mTORC1-GRASP55 hub links nutrient availability and stress signaling to secretory pathway activity and the regulation of the composition of the extracellular proteome.

## Regulation of MTORC1 Activity by Amino Acids

**FIGURE 2:**
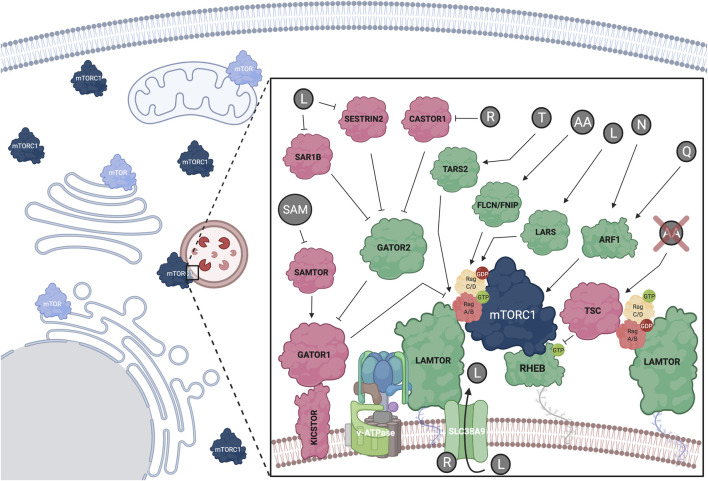
Amino acids regulate mTORC1 at the lysosomal surface via a complex upstream regulatory protein network that impinges upon the Rag GTPase dimers. Positive mTORC1 regulators shown in green; negative regulators shown in red. Additional subcellular locations, where mTOR was previously found, are also depicted. See text for details. Created with BioRender.com

The availability of amino acids (AA), the building blocks of proteins, is presumably the strongest stimulus that activates mTORC1. Already in the 90’s, early studies identified key regulators of protein synthesis, such as S6K1 and 4E-BP1, to be responsive to AA sufficiency, thus linking AA to mTORC1 activation (Blommaart et al., 1995; Hara et al., 1998). However, it was not until 2008 that two groups independently discovered the Rag (Ras-related GTP-binding protein) GTPases as a major hub in AA-sensing by mTORC1 (Kim et al., 2008; Sancak et al., 2008) (Figure 2).

### The Lysosomal Rag GTPases in Amino Acid Sensing and mTORC1 Regulation

The Rag GTPases (hereafter referred to as Rags) form obligate heterodimers, with RagA or RagB binding to RagC or RagD (Sekiguchi et al., 2001), that reside on the lysosomal surface. Because the Rags do not harbor a lipid modification, they are tethered to the lysosomal surface indirectly, via protein-protein interactions with the lysosomal LAMTOR (late endosomal/lysosomal adaptor and MAPK and mTOR activator) complex (also known as “Ragulator”) (Sancak et al., 2010). AA sufficiency induces activation of the Rag dimer, with RagA/B being bound to GTP and RagC/D being bound to GDP (Kim et al., 2008; Sancak et al., 2008; Shen et al., 2017; Lawrence et al., 2018) (Figure 2). Mechanistically, active Rags bind RAPTOR with higher affinity to promote lysosomal mTORC1 recruitment (Sancak et al., 2008). Recent structural studies identified a “claw”-shaped region on RAPTOR via which it recognizes the active state of the Rags (Anandapadamanaban et al., 2019; Rogala et al., 2019). Notably, the Rag-RAPTOR interaction and the lysosomal recruitment of mTORC1 are not sufficient to promote its activation, but rather allow mTORC1 to encounter another small GTPase, its direct activator RHEB (Ras homolog enriched in brain). Accordingly, RHEB depletion inhibits mTORC1 activation by AA without delocalizing it away from lysosomes, thus highlighting the existence of two interconnected mechanistic branches in mTORC1 activation: its lysosomal recruitment by the Rags and its direct activation by RHEB (Sancak et al., 2008; Sancak et al., 2010). Of note, RagA knockout mice die at embryonic day E10.5, underscoring the importance of proper lysosomal mTORC1 regulation in mouse development (Efeyan et al., 2014). Additionally, mice harboring a constitutively active RagA mutant allele, thus being unable to properly inactivate mTORC1 and activate autophagy, die neonatally, further suggesting that fine-tuning of the mTORC1 signaling pathway in response to AA signaling is essential for organismal homeostasis (Efeyan et al., 2013).

### Role of the Rag GTPases and TSC Relocalization in mTORC1 Inactivation Upon Starvation

**FIGURE 3:**
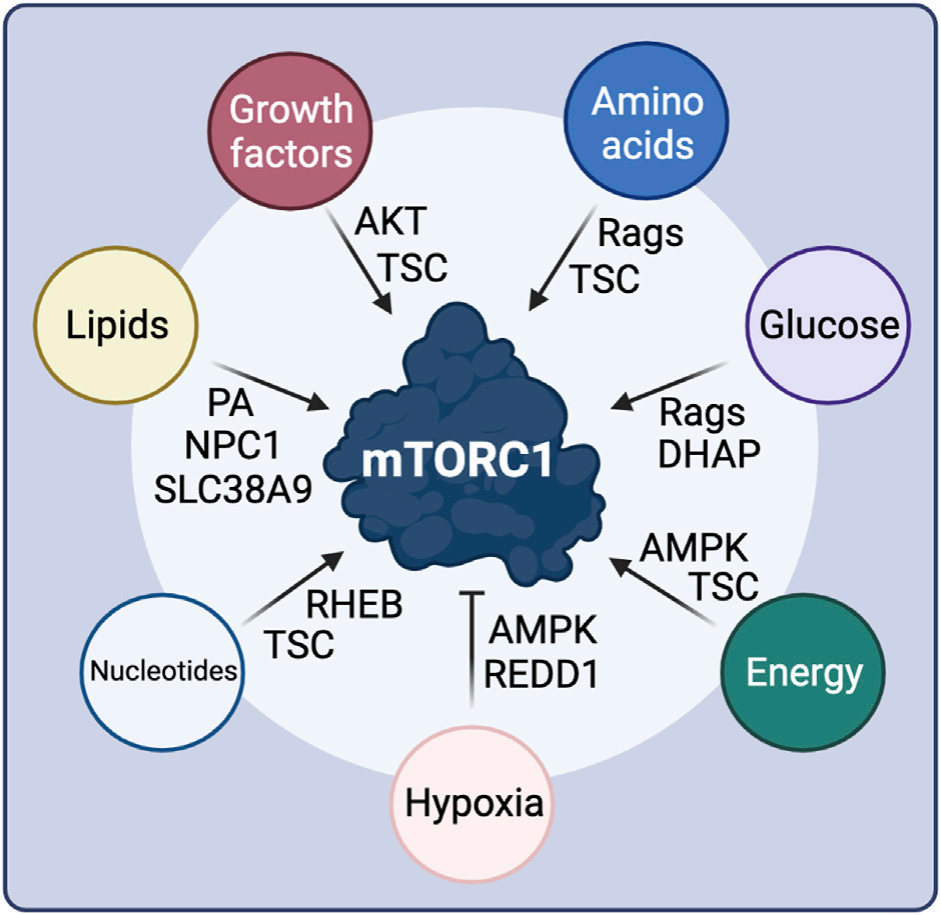
Intra- and extra-cellular stimuli control mTORC1 activity through diverse signaling cascades and upstream mTORC1 regulatory proteins. See text for details. Created with BioRender.com

The main upstream negative regulator of mTORC1 is the heterotrimeric Tuberous Sclerosis Complex (TSC), which is composed of TSC1 (or hamartin), TSC2 (or tuberin) and TBC1D7 (TBC1 domain family member 7) (Dibble et al., 2012; Yang et al., 2021). As discussed more extensively in the following sections, TSC functions by inactivating the direct mTORC1 activator, RHEB (Figure 2). Nearly all stimuli that regulate mTORC1, including AA, signal at least in part via the TSC (Demetriades et al., 2016) (Figure 3). Previous work from our group and others revealed that ΑΑ removal inactivates mTORC1 via the lysosomal relocalization of the TSC complex, and that the TSC is necessary for proper mTORC1 inactivation and cellular response to AA starvation (Demetriades et al., 2014; Carroll et al., 2016). Together with additional lysosomal factors (discussed below), the Rags play a key role in the recruitment of TSC to lysosomes. Lowering Rag expression or preventing the starvation-induced inactivation of the Rag dimer (e.g., by knocking-down GATOR1 components) was able to prevent accumulation of TSC to lysosomes upon AA deprivation (Demetriades et al., 2014). Accordingly, decreasing Rag levels in *Drosophila* or mammalian cells makes them partially insensitive to AA removal due to the defective recruitment of the TSC (Demetriades et al., 2014). Therefore, in addition to regulating mTORC1 re-activation in response to AA resupplementation, the Rags are also actively involved in the inhibition of mTORC1 in starved cells.

Follow-up work suggested that the lysosomal relocalization of TSC is not restricted to AA starvation, but is rather a universal response to cellular stresses; each individual stress stimulus, when applied singly to cells, is sufficient to drive the accumulation of TSC to the lysosomal surface to inhibit mTORC1 (Menon et al., 2014; Demetriades et al., 2016). Although this response is a shared characteristic between multiple stresses, each stress seemingly leads to TSC relocalization via diverse signaling cascades, with growth factor signaling causing the Akt (also known as PKB, protein kinase b)-mediated phosphorylation of TSC2 (Menon et al., 2014), whereas hyperosmotic stress leads to the activation of a calyculin-A-sensitive phosphatase and various kinases impinging upon TSC2 to regulate TSC localization (Plescher et al., 2015).

Interestingly, some studies suggested that—besides the Rag-TSC interaction—TSC binding to membrane-bound RHEB is also contributing to its recruitment to lysosomes upon growth factor or arginine removal (Menon et al., 2014; Carroll et al., 2016). More recent work has revealed two additional tethering mechanisms via which TSC is recruited to lysosomes to inhibit mTORC1 in response to simultaneous removal of AA and growth factors: the G3BP1/2 (Ras GTPase-activating protein-binding proteins 1 and 2) proteins, which are canonically involved in stress granule formation (Prentzell et al., 2021) and recruit the TSC via interacting with the TSC2 subunit; and the lysosomal PI3,5P_2_ (phosphatidylinositol 3,5-biphosphate) lipids that facilitate TSC tethering via interactions with a positively charged region at the N-terminus of TSC1 (Fitzian et al., 2021). Importantly, these studies demonstrate that the inactivation of mTORC1 is not a passive process, due to the absence of positive signals, but rather involves the active participation of key cellular mechanisms, such as the nutrient starvation- and stress-induced lysosomal relocalization of the TSC complex. Notably, the mechanistic aspects of signal integration on TSC, of its recruitment to the lysosomal surface, and of the cellular adaptation to stress are not yet fully understood.

### Regulation of Rag GTPase Activity by Amino Acids

Following the initial discovery of the Rags as key players in AA sensing and mTORC1 regulation, a complex upstream regulatory protein network that signals AA sufficiency to regulate their activity was identified (Figure 2). Typically, guanine exchange factors (GEFs) or GTPase activating proteins (GAP) regulate the activity of small GTPases via promoting their binding to GTP or catalyzing GTP-to-GDP hydrolysis, respectively. The pentameric LAMTOR complex was the first identified GEF for RagA/B and their interaction was shown to be dependent on AA and the v-ATPase (Zoncu et al., 2011; Bar-Peled et al., 2012). A later study identified SLC38A9 (sodium-coupled neutral amino acid transporter 9), an AA transporter that resides on the lysosomal surface as a RagA GEF (Shen and Sabatini, 2018). In contrast, the trimeric GATOR1 protein complex consisting of DEPDC5 (DEP domain-containing 5), NPRL2 (nitrogen permease regulator-like 2), and NPRL3 (nitrogen permease regulator-like 3) functions as a GAP for RagA, and promotes GTP hydrolysis upon AA insufficiency via a poorly understood mechanism (Bar-Peled et al., 2013; Shen et al., 2018). In turn, GATOR1 acts downstream of a protein complex named GATOR2, that is composed of MIOS (meiosis regulator for oocyte development), WDR24 (WD repeat domain 24), WDR59 (WD repeat domain59), SEH1L (Seh1-like nucleoporin), and SEC13 (Sec13 homolog nuclear pore and COPII coat complex component) and is required for mTORC1 activity. GATOR1 lysosomal recruitment was also found to be regulated by KICSTOR, a protein complex comprised of KPTN (Kaptin), ITFG2 (integrin-α FG-GAP repeat containing 2), C12orf66, and SZT2 (seizure threshold 2), which bind GATOR1 and facilitate its interaction with both the Rags and GATOR2 (Peng et al., 2017; Wolfson et al., 2017). Additionally, SH3BP4 (SH3 domain-binding protein 4) was shown to be a negative regulator of the Rags by interacting with the inactive dimer and preventing its activation during AA starvation (Kim Y.-M. et al., 2012).

Although the GEF for RagC/D is still unknown, the FLCN (Folliculin)-FNIP1/2 (FLCN-interacting proteins 1 and 2) complex was described as a GAP for these Rags (Petit et al., 2013; Tsun et al., 2013). Because, in the active Rag dimer, RagC/D is GDP bound, the FLCN-FNIP complex functions as a positive regulator of mTORC1 in response to AA. Of note, FLCN is mutated in Birt-Hogg-Dubé syndrome patients, where RagC/D activity and downstream regulation of TFEB/TFE3 seem to be particularly affected, highlighting FLCN-Rag-mTORC1-TFEB as an important signaling axis in human disease (Napolitano et al., 2020). Interestingly, in cells with inactive mTORC1, nuclear TFEB upregulates RagD gene expression, thus forming a regulatory loop that facilitates fine-tuning of mTORC1 activity in response to AA availability (Di Malta et al., 2017).

Several other proteins have been previously shown to respond to AA availability to regulate mTORC1 via the Rags, with the precise mechanistic details being less clear for some of those. For instance, MAP4K3 (mitogen-activated protein kinase 3) was shown to participate in AA signaling upstream of RagC/D (Findlay et al., 2007; Yan et al., 2010). Another seemingly important factor for mTORC1 activation by AA is SQSTM1 (sequestosome1; or p62), which interacts with both RAPTOR and the Rags to facilitate lysosomal recruitment of mTORC1 when AA are present (Duran et al., 2011). Moreover, SQSTM1 acts in coordination with TRAF6 (TNF receptor associated factor 6) in the K63-linked ubiquitination of mTOR, which is also involved in regulating lysosomal translocation of mTORC1 (Linares et al., 2013). Finally, GPR137B (G protein-coupled receptor 137B) was recently shown to positively regulate mTORC1 via binding to RagA (Gan et al., 2019), presumably regulating its activity and lysosomal localization, which is in line with previous findings showing that the Rags cycle on and off the lysosomal surface in response to AA availability (Lawrence et al., 2018). Future work will be important to disentangle how this diverse panel of proteins act coordinately to signal AA sufficiency to the Rags, and what is the relative contribution of each of them in mTORC1 signaling, in response to different nutritional conditions and in different cell types.

### Direct Amino Acid Sensors Upstream of the Rag GTPases

Several studies over the last decade have identified a number of lysosomal or cytoplasmic proteins that function as direct sensors for specific individual AA (or groups of AA) and transmit information about their availability to control Rag activity and lysosomal recruitment of mTORC1 (Figure 2). Notably, this striking specificity for certain AA suggested the existence of multiple parallel routes via which AA regulate mTORC1.

*Arginine sensing:* SLC38A9 is a neutral amino acid transporter that acts as a lysosomal arginine sensor. Upon binding to arginine, SLC38A9 activates mTORC1 in a v-ATPase- and LAMTOR-dependent manner (Jung et al., 2015; Rebsamen et al., 2015; Wang et al., 2015; Wyant et al., 2017). Interestingly, SLC38A9 is important for the arginine-dependent export of leucine from the lysosomal lumen, highlighting the crosstalk between AA that activate mTORC1 (Wyant et al., 2017). Additionally, arginine can be sensed by the cytoplasmic sensors CASTOR1/2 (cytosolic arginine sensor for mTORC1), which bind and inhibit GATOR2 when arginine levels are low, thus inactivating mTORC1. When arginine is abundant, its binding to CASTOR1/2 weakens the inhibitory CASTOR-GATOR2 interaction, leading to mTORC1 activation (Saxton et al., 2016a; Chantranupong et al., 2016).

*Leucine sensing:* Similar to the CASTOR proteins in arginine sensing, SESTRIN proteins bind and inactivate GATOR2 in leucine-deprived cells (Chantranupong et al., 2014; Parmigiani et al., 2014). Leucine binding to SESTRIN2 relieves GATOR2 inhibition, which can then signal to activate mTORC1 via the Rags (Saxton et al., 2016b; Wolfson et al., 2016). Interestingly, SESTRINs were originally discovered as stress response proteins (Budanov et al., 2002) and were also identified to negatively regulate mTORC1 via the AMPK-TSC2 axis (Budanov and Karin, 2008), suggesting that they are part of a broader cellular response to suboptimal growth conditions (Lee et al., 2016). A recent study identified SAR1B (secretion associated Ras-related GTPase 1B) as an independent leucine sensor that acts in the same way as SESTRINs to regulate mTORC1 (Chen et al., 2021). Additionally, leucine can also activate mTORC1 via its aminoacyl-tRNA synthetase, LARS (leucyl-tRNA Synthetase 1), which acts as a GAP for RagD (Han et al., 2012). More recently, an alternative sensing mechanism for leucine was proposed based on acetyl-CoA, a downstream product in leucine metabolism, hence expanding the spectrum of possible signaling molecules in AA sensing: in this case, acetyl-CoA was shown to signal leucine availability via facilitating RAPTOR acetylation, a modification that led to mTORC1 activation (Son et al., 2019).

*Methionine sensing:* As with leucine, methionine sensing occurs via a downstream metabolite. S-adenosylmethionine (SAM) is a methionine-derived compound that functions as a donor of the methyl group for methylation of cellular proteins and DNA. Opposite to leucine and arginine, SAM does not signal via GATOR2, but instead binds to SAMTOR (S-adenosylmethionine sensor upstream of mTORC1; or C7orf60) to release its interaction with GATOR1 and KICSTOR and to promote mTORC1 activation (Gu et al., 2017).

*Threonine sensing:* Similar to the role of LARS in leucine signaling, threonine levels regulate mTORC1 via TARS2 (mitochondrial threonyl-tRNA synthetase 2). Threonine binding to TARS2 causes its association with GTP-bound RagC, and subsequent activation of the RagA-RagC dimer, leading to activation of mTORC1 (Kim et al., 2021). Because TARS2 seemingly lacks GEF domains, it is unlikely that it acts directly on RagA, raising the possibility that it might act through a so-far-unknown GEF.

*Sensing of other AA:* Glutamine was previously shown to activate mTORC1 in a Rag-dependent manner via α-ketoglutarate, a product of glutaminolysis, which also requires leucine (Duran et al., 2012). In addition to the AA discussed above, alanine, histidine, serine and valine have also been linked to mTORC1 activation (Kobayashi et al., 2014; Dyachok et al., 2016) and were shown to act via Rag-dependent mechanisms (Meng et al., 2020). Interestingly, certain AA seem to play a dominant role over others in the regulation of mTORC1, further highlighting the intricate complexity of the cellular AA sensing network (Dyachok et al., 2016). More studies are required to fully understand the differential activation of mTORC1 by distinct AA, and additional direct AA sensors are likely to emerge in the near future.

### Rag-independent Regulation of mTORC1 by Amino Acids

Although most studies investigating how AA regulate mTORC1 have focused on the Rag-dependent upstream signaling network, a growing body of evidence in the recent years hints for the existence of additional Rag-independent mechanisms, via which AA control mTORC1 activity (Figure 2). Notably, in RagA/B double-knockout cardiomyocytes that show impaired lysosomal function, phosphorylation of mTORC1 substrates, such as 4E-BP1, is not significantly affected (Kim et al., 2014). Similar observations were drawn from work using RagA-mutant zebrafish (Shen et al., 2016) and Rag knockout or knock-down cells (Demetriades et al., 2014; Efeyan et al., 2014; Kim et al., 2014; Jewell et al., 2015; Shen et al., 2016). Moreover, glutamine resupplementation was shown to reactivate mTORC1 independently from the Rag GTPases, via inducing its lysosomal relocalization in an Arf1 (adenosine diphosphate ribosylation factor-1)- and v-ATPase-activity-dependent manner (Jewell et al., 2015). A follow-up study suggested the involvement of PLD1 (Phospholipase D1)—an enzyme previously linked to mTOR complex stability and activity, via the production of phosphatidic acid (PA) (Toschi et al., 2009; Yoon et al., 2011)—and of α-ketoglutarate in the Arf1-dependent, Rag-independent mTORC1 activation (Bernfeld et al., 2018). Interestingly, the Rag-independent mode of glutamine signaling to mTORC1 seems to be conserved also in yeast lacking expression of Gtr (GTP-binding protein GTR) proteins, the Rag orthologs (Stracka et al., 2014). However, Pib2 (phosphatidylinositol 3-phosphate-binding protein 2), the proposed glutamine sensor in yeast (Ukai et al., 2018), is not conserved in humans, and the closest candidates Phafin1 or R3HCC1 (R3H and coiled coil domain–containing protein 1) did not affect glutamine signaling to mTORC1 (Meng et al., 2020). Since glutamine is one of the most important AA to fuel cell growth and metabolism, it is of utmost importance to understand the precise mechanisms by which this AA controls the master regulator of cell growth, mTORC1. A recent study showed that, besides glutamine, also asparagine can activate mTORC1 in Rag-mutant cells via Arf1 (Meng et al., 2020). It should be noted, however, that the mechanism via which the Golgi-localized Arf1 GTPase influences the lysosomal recruitment and reactivation of mTORC1 remains elusive. Considering the more general role of Arf1 in ER-Golgi vesicle trafficking, a more indirect mechanism involving the maturation of the endo-lysosomal machinery cannot be excluded, based on the currently available data.

### mTORC1 Regulation at Distinct Cellular Locations

Although the lysosomal surface has emerged as the main subcellular location for mTORC1 activation by AA (Sancak et al., 2010), this view has been expanded in the recent years to include additional compartments (summarized in Betz and Hall (2013)) (Figure 2). Interestingly, roughly half of mTOR does not colocalize with lysosomes (Lawrence et al., 2018) and has been found on other organelles, such as the Golgi, mitochondria, ER (endoplasmic reticulum), and in the nucleus (Schieke et al., 2006; Liu and Zheng, 2007; Ramanathan and Schreiber, 2009; Yadav et al., 2013; Giguere, 2018; Gosavi et al., 2018). Similarly, many of the upstream mTORC1 regulators and downstream substrates are either non-lysosomal or only partially lysosomal proteins. For instance, RHEB, the indispensable direct mTORC1 activator, is also found to be enriched in many different endomembranes besides lysosomes, including the Golgi, ER and peroxisomes (Buerger et al., 2006; Hanker et al., 2010; Yadav et al., 2013; Zhang et al., 2013; Gosavi et al., 2018; Hao et al., 2018; Angarola and Ferguson, 2019). Moreover, TSC2, the main negative regulator of mTORC1, has been shown to accumulate in non-lysosomal structures, some of which can be peroxisomes (Zhang et al., 2013; Demetriades et al., 2014). As also mentioned above, Arf1 seems to regulate mTORC1 activity independently from the Rags, functioning from the Golgi (Jewell et al., 2015). Finally, mTORC1 substrates, such as S6K1 and 4E-BP1, are cytoplasmic components of the translation initiation machinery, further supporting a model for active mTORC1 also away from the lysosomal surface (Holz et al., 2005; Zhou et al., 2015; Ahmed et al., 2019). Interestingly, a recent study found mTORC1 partially colocalizing with focal adhesions (FA), where it relocalizes via peripheral lysosome distribution. Because growth factor (GF) receptors and AA transporters also show local accumulation at FA, the presence of mTORC1 at these structures highlights them as key signaling hubs for mTORC1 activation by both GF and AA (Rabanal-Ruiz et al., 2021). Collectively, these findings suggest that the regulation of mTORC1 by AA is broader than previously described, and likely involves additional, currently unknown regulatory factors, mechanisms, and subcellular compartments.

## Regulation of MTORC1 by Other Physiological Stimuli

### Growth Factor and Cytokine Signaling

The regulation of mTORC1 by GF signaling is largely mediated by the TSC protein complex (Tee et al., 2002) (Figure 3). Mutations in *TSC1* or *TSC2* lead to the development of tuberous sclerosis, a disease characterized by multiple benign tumors in several organs, hinting to its function in the growth-related mTORC1 pathway (Huang and Manning, 2008). TSC2 acts as a GAP towards RHEB, and inactivates it by promoting the conversion of RHEB-GTP to RHEB-GDP, thus also inactivating mTORC1 (Tee et al., 2003b; Garami et al., 2003). TSC activity is regulated via multi-site phosphorylation of TSC2 by Akt, which is in turn controlled directly by GF availability (Inoki et al., 2002; Manning et al., 2002; Tee et al., 2003a). Growth factor binding to the respective GF receptor at the cell surface promotes the activation of PI3K (phosphoinositide 3-kinase) and PDK1, with the latter directly phosphorylating Akt (Hoxhaj and Manning, 2020). Moreover, mTORC2 phosphorylates a different residue on Akt for maximal Akt activity, thus establishing a crosstalk between the two mTOR complexes (Sarbassov et al., 2005). Akt-dependent phosphorylation of TSC2 promotes its dissociation from the lysosomal surface where RHEB also resides, thereby releasing RHEB inhibition and promoting mTORC1 activity (Menon et al., 2014; Plescher et al., 2015; Demetriades et al., 2016). Furthermore, active Akt can also activate mTORC1 directly via phosphorylating PRAS40 and releasing its endogenous inhibitory function within the complex. Notably, active mTORC1-S6K1 signaling forms a negative feedback loop in the GF signaling pathway, through an S6K1-dependent inhibitory phosphorylation event on IRS-1 (insulin receptor substrate 1), which is important for fine-tuning the cellular response to GF and in the development of resistance to mTOR inhibitors (Harrington et al., 2004; Shah et al., 2004).

An additional branch in the GF signaling pathway also acts via the TSC to regulate mTORC1. Phosphorylation of TSC2 by ERK (mitogen-activated protein kinase 3) and RSK (p90 ribosomal S6 kinase), downstream of the Ras/receptor tyrosine kinase pathway, leads to TSC inhibition (Roux et al., 2004; Ma et al., 2005). In addition to the canonical GF-dependent TSC inactivation, TSC2 is also phosphorylated and inhibited by GSK3β (glycogen synthase kinase-3 beta), a component on the Wnt signaling pathway, which increases mTORC1 activity (Inoki et al., 2006). Unlike most phosphorylation events that take place on TSC2, IKKβ (inhibitor of nuclear factor kappa-B kinase subunit beta) directly phosphorylates the TSC1 component in response to TNFα stimulation (Lee et al., 2008), whereas CDK1 (cyclin-dependent kinase 1) couples cell growth to cell cycle control via TSC1 phosphorylation (Astrinidis et al., 2003).

### Energy, Glucose and Oxygen Availability

As cell growth is a highly energy consuming cellular process, mTORC1 activity also responds to fluctuations in energy availability (Figure 3). The major component of the cellular energy sensing machinery is the protein kinase AMPK (5′-AMP-activated protein kinase; also known as PRKAA1), which responds to AMP when cells confront energetic stress, e.g., upon nutrient (AA or glucose) starvation, or under low oxygen conditions (Herzig and Shaw, 2018). Therefore, AMPK functions as a negative regulator of mTORC1, linking the cellular energy status to downstream metabolic functions. At the molecular level, AMPK inhibits mTORC1 by directly phosphorylating and activating TSC2 upstream of mTORC1 (Inoki et al., 2003), as well as by phosphorylating RAPTOR within the mTOR complex (Gwinn et al., 2008). Glucose seems to regulate mTORC1 via multiple different pathways, some of which may also be linked to each other (Figure 3). While initial studies showed that glucose starvation inhibits mTORC1 through AMPK activation, others suggested that glucose also regulates mTORC1 activity and lysosomal localization in a Rag-dependent manner, even in AMPK knockout cells (Efeyan et al., 2013). A recent follow-up study identified the glycolysis intermediate DHAP (dihydroxyacetone phosphate) as a metabolite that activates mTORC1 independently from AMPK, when glucose is present, although the direct sensor remains elusive (Orozco et al., 2020). Whether DHAP controls mTORC1 activity upstream of the Rags or in a Rag-independent manner is also unclear (Orozco et al., 2020). Finally, low glucose levels are also sensed by mTORC1 via directly interacting with the glycolytic enzyme hexokinase-II (HK-II) (Roberts et al., 2014).

Hypoxia also acts independently from AMPK to suppress mTORC1 activity (Arsham et al., 2003), via upregulating REDD1 (regulated in development and DNA damage responses 1) levels (Figure 3). In normoxic conditions, TSC2 is inactivated via binding to 14-3-3 proteins. REDD1 binding to 14-3-3 proteins releases TSC2, which in turn inhibits mTORC1 (Brugarolas et al., 2004; DeYoung et al., 2008).

### Nucleotides and Lipids

Concomitantly with mTORC1 controlling nucleotide and lipid biosynthesis, the levels of these macromolecules signal to regulate mTORC1 activity (Figure 3). Purine nucleotide availability was reported to activate mTORC1 either by influencing RHEB farnesylation and membrane association (Emmanuel et al., 2017), or by inhibiting TSC activity (Hoxhaj et al., 2017). Lipids were also proposed to directly activate mTORC1, for instance via the generation of PA (Menon et al., 2017), which is essential for the stability and activity of both mTORC1 and mTORC2 (Toschi et al., 2009). PA was also recently shown to play a role in the lysosomal recruitment of mTORC1 (Frias et al., 2020). Additionally, over the past years, an increasing body of evidence showed that cholesterol signals directly to activate mTORC1 at the lysosomal surface, via a mechanism that involves SLC38A9 (sodium-coupled neutral amino acid transporter 9) and the NPC1 cholesterol transporter (Castellano et al., 2017; Lim et al., 2019; Davis et al., 2021).

In conclusion, these studies highlight the dual role of mTORC1 both as a regulator of virtually all cellular biosynthetic processes and as a sensor of key metabolites and nutrients that are produced from these metabolic pathways. The existence of such intricate autoregulatory loops allows for proper control of metabolism and homeostasis in cells.

## Role of MTORC1 Signaling and Downstream Functions in Aging

During aging, cellular and organismal physiology are progressively dysregulated, increasing susceptibility to diseases and, ultimately, leading to death. A key hallmark of aging is deregulated nutrient sensing, which is mainly reflected in mTORC1 signaling (Lopez-Otin et al., 2013). In turn, by regulating the majority of cellular functions, mTORC1 impacts most other hallmarks of aging, thus highlighting its central role in the control of organismal homeostasis (Papadopoli et al., 2019). The spectrum of mTORC1 downstream functions that are dysregulated upon aging underscores the complex nature of the aging process itself, and complicates our understanding about the intricate interplay between these cellular activities.

### Dysregulation of Protein Synthesis

Proteins are the main effectors of cellular functions; hence, it is of extreme importance that both protein synthesis and protein quality control systems work flawlessly for cells to function properly. It is now well-established that, with aging, protein synthesis becomes deregulated, both in quantitative and qualitative terms, and that this dysfunction is a major contributor to age-related maladies (Tavernarakis, 2008; Steffen and Dillin, 2016; Gonskikh and Polacek, 2017; Anisimova et al., 2018).

**FIGURE 4:**
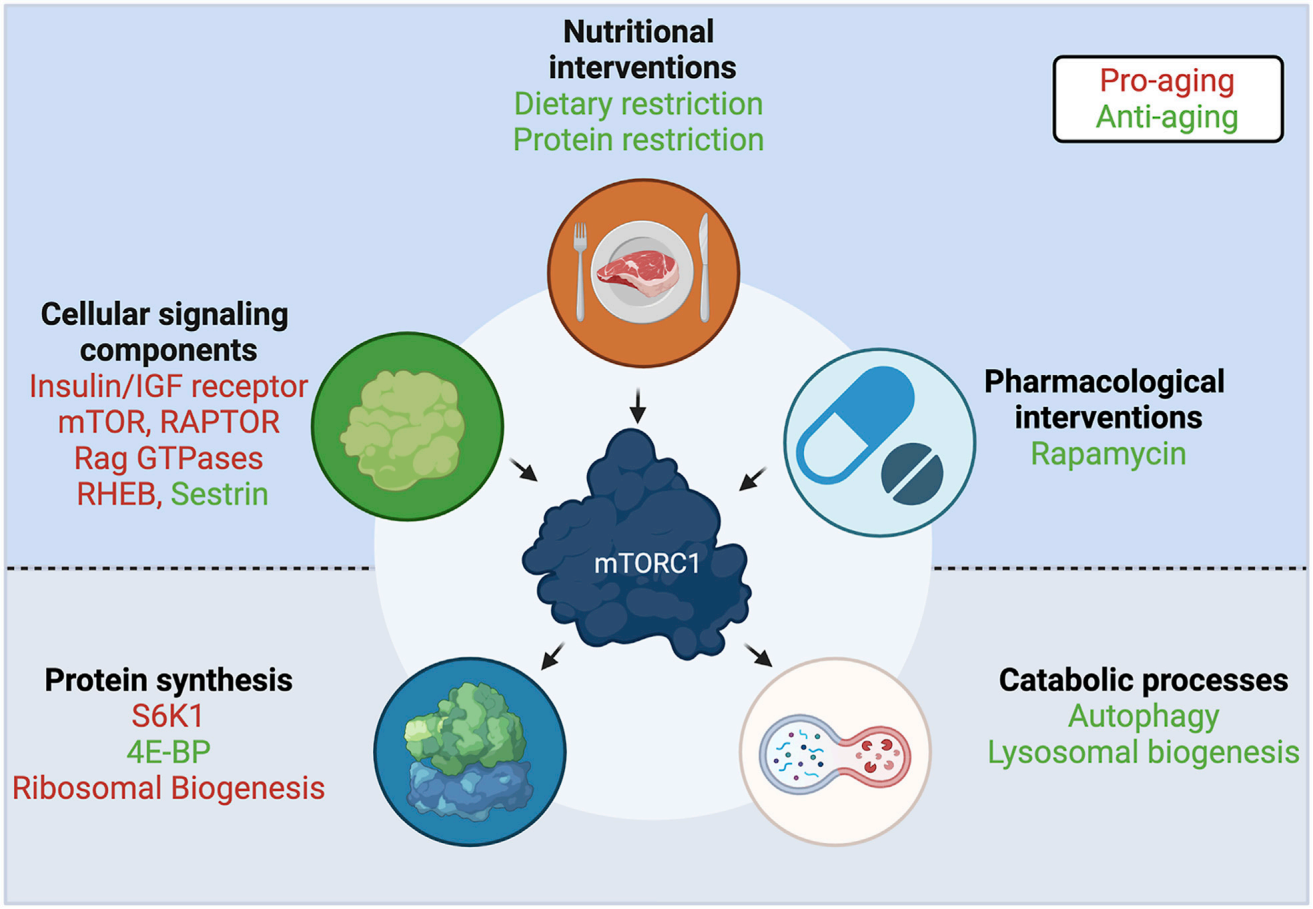
A central role for mTORC1 in aging. Interventions and upstream cellular components that regulate mTORC1 activity, and downstream effectors and functions that are regulated by mTORC1, participate in the aging process. Components with anti-aging properties shown in green; components with pro-aging properties shown in red. See text for details. Created with BioRender.com

Since protein synthesis is a key function regulated by mTORC1, several approaches to understand the role of mTORC1 in aging have focused on the known translation-related effectors downstream of mTORC1 (Figure 4). Accordingly, the lifespan extension in fruit flies treated with Rapamycin to inhibit TORC1, fully depends on both S6K1 and 4E-BP (Bjedov et al., 2010). Further confirming the importance of the S6K signaling branch in aging, mutation of the yeast (Sch9; Serine/threonine-protein kinase SCH9) (Kaeberlein et al., 2005), *C. elegans* (rsks-1; ribosomal protein S6 kinase) (Hansen et al., 2007; Pan et al., 2007), *Drosophila* (dS6K; ribosomal protein S6 kinase) (Kapahi et al., 2004), or mouse S6K1 homologs (Selman et al., 2009), recapitulated most of the lifespan extension effects observed in the respective mTOR mutants. Similarly, in *Drosophila*, dietary restriction resulted in increased levels of the translation inhibitor 4E-BP (Zid et al., 2009). The importance of 4E-BP-mediated translation inhibition is also evident in *C. elegans*, where interventions that decrease eIF4E-dependent translation have also been linked to lifespan extension (Hansen et al., 2007; Pan et al., 2007; Syntichaki et al., 2007). Moreover, 4E-BP expression in *Drosophila* muscles is able to improve systemic proteostasis and promote lifespan extension (Demontis and Perrimon, 2010), in line with a later mouse study showing that 4E-BP1 activity in muscles influences organismal metabolism during aging (Tsai et al., 2015). Collectively, these studies show that the modulation of the translational machinery downstream of mTORC1 is a conserved process that is tightly linked to aging.

In the search for a convergent mechanism among known lifespan extension interventions, including TOR activity modulation, nucleolar size—an indicator of ribosomal biogenesis—was found as a common evolutionarily-conserved marker, with smaller nucleoli correlating with increased longevity in worms, mice, fruit flies, and humans (Tiku et al., 2017; Tiku and Antebi, 2018) (Figure 4), and nucleolar size correlating with donor age in healthy individuals (Buchwalter and Hetzer, 2017). Moreover, a model of Hutchinson-Gilford progeria syndrome (HGPS) shows larger nucleoli, increased ribosomal biogenesis and an overall increase in protein translation levels (Buchwalter and Hetzer, 2017), thus ascribing a potential functional connection between this aging biomarker and the global increase in protein synthesis that is observed over aging. These findings suggest that, beyond the direct regulation of multiple translational components by mTORC1, the translational machinery is functionally interconnected at different levels. Untangling the causal relationship between these individual links will be an important milestone in our understanding of how interventions that extend lifespan work at the molecular level.

### Dysregulation of Autophagy

Aging is accompanied by a progressive functional decline in the cellular protein quality control and organelle degradation machineries. Autophagy is a central process in the clearance of damaged cellular components, as well as in the mobilization and recycling of cellular nutrients, therefore its malfunction directly impairs organismal homeostasis (Leidal et al., 2018). Accordingly, dysregulated autophagy results in the accumulation of protein aggregates and damaged organelles, which is a characteristic of many age-associated diseases such as Alzheimer’s (Leidal et al., 2018; Barbosa et al., 2019). In fibroblasts, constitutive mTORC1 activation upon aging also disturbed autophagy, while rapamycin treatment potently reversed this effect (Romero et al., 2016) (Figure 4). Generally, autophagic function and the expression of autophagy-related genes (ATGs), in particular, are downregulated upon aging in many organisms, such as in *C. elegans* (Chang et al., 2017), *Drosophila* (Simonsen et al., 2008), rodents (Donati et al., 2001; Del Roso et al., 2003; Kaushik et al., 2012), and humans (Lipinski et al., 2010). Consequently, approaches aiming to restore autophagy are frequently investigated in the context of lifespan extension studies. For instance, autophagy activation by overexpression of a single autophagic protein, ATG5, was shown to extend lifespan in mice (Pyo et al., 2013). Moreover, a Beclin1 mutant knock-in mouse model, in which basal autophagy is enhanced, demonstrated increased lifespan and healthspan (Fernandez et al., 2018). Similar approaches that aim to increase autophagic levels, as a means to extend lifespan, were also employed in other model organisms, including *C. elegans* (Melendez et al., 2003; Kumsta et al., 2019) and *Drosophila* (Simonsen et al., 2008; Aparicio et al., 2019; Bjedov et al., 2020). The fact that most of the lifespan extending interventions that target mTORC1 were also shown to influence autophagy, as observed in yeast, *C. elegans*, and *Drosophila* studies (Jia and Levine, 2007; Hansen et al., 2008; Alvers et al., 2009; Bjedov et al., 2010; Matecic et al., 2010), underscores the importance of fine-tuning autophagy in the aging process.

### Lysosomal Dysregulation

Lysosomes are important cellular organelles that lie both upstream and downstream of mTORC1—being a major site of mTORC1 activation, and of autophagic degradation, respectively. Additionally, mTORC1 controls both lysosomal activity and biogenesis via the phosphorylation of the TFEB/TFE3 transcription factors: mTORC1 inhibition causes TFEB/TFE3 relocalization to the nucleus and induction of a transcriptional program that promotes lysosomal biogenesis and autophagy. In *C. elegans*, nuclear localization of the TFEB homolog, namely hlh-30 (helix-loop-helix protein 30), is a core feature of many lifespan extending interventions (Lapierre et al., 2013), and blocking TFEB/hlh-30 nuclear export was shown to be sufficient for lifespan extension (Silvestrini et al., 2018). Likewise, livers of dietary-restricted mice show increased nuclear TFEB, suggesting that the beneficial effects of TFEB activation are conserved through evolution (Lapierre et al., 2013). Further supporting a link between TFEB function and healthy aging, Alzheimer’s and ALS (amyotrophic lateral sclerosis) patients have an overall decrease of nuclear TFEB (Wang et al., 2016).

Because lysosomal degradation is the last step in the autophagic process, it is not surprising that lysosomal function is also impaired in aging cells, as observed in the yeast vacuole (Hughes and Gottschling, 2012) and in *C. elegans* lysosomes (Folick et al., 2015; Sun et al., 2020). In yeast, changes in vacuolar pH seemingly constitute an important factor for the decline in its function, with elevated pH values observed upon aging (Hughes and Gottschling, 2012). This increase influences the ability of vacuoles to store AA, potentially resulting in a toxic accumulation of cytosolic AA (Hughes and Gottschling, 2012). Accordingly, in human leukocytes, the expression of LAMP2 (lysosome-associated membrane protein 2), an important protein for lysosomal function (Eskelinen et al., 2002) and autophagosome-lysosome fusion (Saftig et al., 2008) also decreases with age (Huang et al., 2012). Highlighting its role in lifespan extension, improvement of lysosomal function is also a common feature of many lifespan extending interventions (Sun et al., 2020). For instance, methionine restriction in yeast enhances autophagy at least in part via modulating vacuolar pH (Ruckenstuhl et al., 2014). The identification of lysosomes as signaling platforms, in conjunction with their role as the main cellular recycling organelles, establishes them as a central point for interventions targeting aging.

Our current understanding of the aging process positions mTORC1 simultaneously as a gate-keeper of cellular physiology and as the main driver of most age-associated pathological alterations. Whether dysregulation of mTORC1 over aging is the trigger for the malfunction of key cellular processes, such as translation, autophagy, and lysosomal biogenesis, or vice versa is an important question that remains to be elucidated.

## Interventions Targeting mTORC1 to Tackle Aging

Due to the central role of mTORC1 and the importance of associated downstream functions in cellular and organismal aging, genetic, nutritional, and pharmacological anti-aging interventions commonly target the mTORC1 pathway, aiming to reverse deregulated homeostatic processes.

### Genetic Perturbations in mTORC1 Signaling Components

Similar to the mTORC1 effectors described above, components of this complex, as well as some of its upstream regulators, were also shown to play an important role in the aging process (Figure 4). Not unexpectedly, downregulation of the *C. elegans* mTOR homolog, let-363 (Vellai et al., 2003; Hansen et al., 2007), or the RAPTOR homolog, daf-15 (Jia et al., 2004; Robida-Stubbs et al., 2012) robustly increase lifespan. Notably, human studies suggested that the offspring of nonagenarians show reduced RAPTOR expression in blood cells, which hints that having less mTORC1 is beneficial for human lifespan (Passtoors et al., 2013). Consistently, hypomorphic mTOR mice, that express approximately a quarter of wild-type mTOR levels, live longer and exhibit improved aging tissue biomarkers, compared to their wild-type counterparts (Wu J. J. et al., 2013). Additionally, RNAi of the essential TOR activator Rheb/rheb-1 also led to increased lifespan in *C. elegans* (Robida-Stubbs et al., 2012). In line with their role in AA sensing, the longevity effects that are achieved by protein or AA restriction in *C. elegans* can also be recapitulated by mutations or decreased expression of the RagA/RagC homologs, namely raga-1/ragc-1 (Schreiber et al., 2010; Robida-Stubbs et al., 2012). Similarly, the leucine sensor SESTRIN was shown to be necessary for the dietary-restriction-induced lifespan extension in *Drosophila*, while a constitutively active SESTRIN mutant, that cannot bind leucine, downregulates TORC1 activity and is sufficient to promote longevity even in fed flies (Lu J. et al., 2020). The study by Lu et al. suggests that individual AA sensors may be promising candidates for the development of compounds that target specific branches of mTORC1 signaling. Finally, perturbations in TSC, a shared component of the growth factor and AA sensing machineries, were shown to modulate aging in model organisms: flies overexpressing dTSC1 and dTSC2 live longer, due to decreased TORC1 activity (Kapahi et al., 2004); and proper TSC function is essential for the beneficial effects of protein restriction in mice (Harputlugil et al., 2014), further supporting a key role for TSC in aging and the response to nutrients.

### Perturbations in the Insulin/IGF Signaling Pathway

The first longevity-related phenotype was described in the late 1980’s in *C. elegans*: worms harboring mutations in the PI3K homologous gene showed increased lifespan (Friedman and Johnson, 1988). PI3K is a kinase activated upon insulin binding to the IR (insulin receptor) or to IGF1R (insulin-like growth factor 1 receptor). Establishing the insulin signaling pathway (IIS) as a major determinant of aging, mutations in daf-2 (the *C. elegans* IR/IGF1R homolog), or InR (the Drosophila IR/IGF1R homolog) also extended lifespan (Kenyon et al., 1993; Tatar et al., 2001). Moreover, heterozygous mice harboring only one allele of the IGF1R gene also live longer (Holzenberger et al., 2003). Remarkably, the effect of the IIS pathway seems to be confirmed also in centenarian humans, where genetic alterations in the IGF1R gene are associated with increased longevity (Suh et al., 2008). Although part of the effects of the IIS pathway is attributed to the regulation of the Akt/FOXO (Forkhead box protein O) branch (Kenyon et al., 1993), many of the IIS-related interventions impinge on mTORC1 to control its activity (Figure 4), further establishing this complex as a central hub in aging.

### Rapamycin Treatment

Rapamycin is a naturally-derived macrolide compound that demonstrates immunosuppressive, chemotherapeutic, and anti-aging effects in model organisms and in humans. Importantly, rapamycin and its analogs are currently being used in the clinic against various cancers and neurological disorders (Benjamin et al., 2011; Li et al., 2014; Arriola Apelo and Lamming, 2016). Rapamycin acts as a specific allosteric inhibitor of mTORC1, the master regulator of cellular and organismal physiology. Mechanistically, rapamycin functions via a family of proteins called FKBPs (peptidyl-prolyl cis-trans isomerases), which bind to mTOR and inhibit its activity in a rapamycin-dependent manner. Consistent with mTORC1 inhibition being a potent lifespan extension approach, rapamycin constitutes one of the best-described anti-aging compounds (Figure 4). Rapamycin treatment increases chronological lifespan in yeast (Powers et al., 2006) and in *Drosophila* (Bjedov et al., 2010). Similarly, mice treated with rapamycin starting at either 9 or 20 months of age showed increased survival (Harrison et al., 2009), demonstrating that pharmacological interventions are a feasible approach to achieve lifespan extension in mammals, and, most importantly, that such treatments could also be initiated later in life. Several follow-up studies, using various drug concentrations and time points, strengthened this notion further (reviewed in Selvarani et al. (2020)). Because of the role of rapamycin as an anti-cancer agent, and the involvement of hyperactive mTORC1 in most cancer types, it was originally assumed that its beneficial effects in aging are attributed largely to its ability to prevent cancer. However, underscoring the multifaceted role of mTORC1 inhibition in aging, a number of studies showed that rapamycin treatment improves multiple aspects of the aging phenotype, besides lowering cancer incidence (Partridge et al., 2020).

In humans, susceptibility to respiratory tract infections (RTIs) increases with age, most likely because of a decline in immune function. Therefore, the fact that rapamycin can also function as an immunosuppressant seemingly contradicts its role as an anti-aging compound. Against this hypothesis, a landmark study showed that rapamycin treatment actually improved the immune response of elderly to vaccination against influenza (Chen et al., 2009). Furthermore, a recent clinical trial that tested the RTB101 mTOR inhibitor, alone or in combination with the rapamycin analog everolimus, showed a significant reduction in the incidence of RTIs in old individuals (Dzankic and Partridge, 2021; Mannick et al., 2021). Despite rapamycin being one of most promising anti-aging interventions, long-term treatment has been associated with significant side effects, such as hyperlipidemia, hypercholesterolemia, hypertriglyceridemia, glucose intolerance and insulin resistance, thus restricting its wide-spread use to tackle aging in humans (Arriola Apelo and Lamming, 2016; Partridge et al., 2020). Of note, most of these side effects were later attributed to the concomitant inhibition of mTORC2, which occurs following chronic mTORC1 inhibition and as a result of rapamycin binding to mTOR, thus affecting mTORC2 formation (Lamming et al., 2012). Treatment regimens that would prevent mTORC2 inhibition, or the use of different rapamycin analogs have been proposed to circumvent these undesired effects (Apelo et al., 2016). Whether rapamycin (or rapalog) administration will prove to be a safe, mainstream way to fight age-related conditions in humans remains to be seen.

### Dietary Restriction

Dietary restriction (DR) is defined as the reduction of the intake of most constituents of a diet, with the exception of vitamins and minerals, without malnutrition. DR has been shown to increase lifespan and healthspan in rodents several decades ago (McCay et al., 1935; Maeda et al., 1985), and is generally considered a prominent evolutionarily-conserved anti-aging strategy, with several studies demonstrating similar beneficial effects in multiple model organisms (reviewed in Fontana et al. (2010), Fontana and Partridge, (2015)), as well as in human healthspan (Mercken et al., 2013). In yeast, *C. elegans* and *Drosophila*, DR did not elicit a further increase in the lifespan extension achieved by downregulation of TOR signaling, suggesting that both perturbations act through the same pathway (Kapahi et al., 2004; Kaeberlein et al., 2005; Hansen et al., 2007) (Figure 4). Interestingly, the Rapamycin-induced lifespan extension was still additive to that caused by DR in *Drosophila*, hinting that DR may be employing additional mechanisms to mTORC1 inhibition to exert its full effect (Bjedov et al., 2010). More recent studies solidified the notion that pharmacological inhibition of mTORC1 by rapamycin does not equal the effect on mTORC1 caused by DR (Unnikrishnan et al., 2020). A plausible explanation is that rapamycin only partially inhibits phosphorylation of certain downstream mTORC1 substrates, with some of the 4E-BP1 phosphorylation sites being largely unaffected (Kang et al., 2013). Although the precise role that the 4E-BP1 phosphorylation code plays in its function is not fully understood, the important role of 4E-BP1 in the translational response downstream of mTORC1 (Morita et al., 2013; Hulea et al., 2018), hints that this may be a key difference between the two conditions.

### Protein and Amino Acid Restriction

Importantly, DR extends lifespan due to the limitation of dietary nutrients, rather than the limitation of calorie intake alone (Mair et al., 2005), with certain diet components playing a stronger role than others. As a rule of thumb, restriction of nutritional proteins (protein restriction, PR), AA groups, or even individual AA, was shown to be more important than restriction of sugars (Mair et al., 2005; Lopez-Torres and Barja, 2008). This is in line with AA being the most robust activator upstream of mTORC1 in cells (Figure 4). Therefore, interventions aiming to restrict specific macronutrients were proposed as a more targeted alternative to DR.

*Studies in yeast:* Specific restriction of methionine is a potent lifespan inducer in yeast (Wu Z. et al., 2013). This effect was shown to depend on the v-ATPase (Ruckenstuhl et al., 2014), which is coupled to yeast TOR activation (Saliba et al., 2018). Removal of asparagine or glutamate from the culture media, or inhibition of the yeast glutamine synthetase, also extended chronological lifespan in yeast (Powers et al., 2006). Similarly, further studies proposed that removal of threonine, valine, or serine promotes lifespan extension, with threonine and valine acting in a TOR-dependent manner (Mirisola et al., 2014).

*Studies in Drosophila:* Work aiming at understanding how DR leads to healthspan and lifespan extension, revealed that PR alone is sufficient to extend lifespan (Mair et al., 2005; Min and Tatar, 2006). Remarkably, resupplementation of essential amino acids (EAA) to dietary-restricted flies was sufficient to abrogate the DR-induced lifespan extension, underscoring EAA as a major determinant of the DR effect *in Drosophila* (Grandison et al., 2009). In addition, maximum lifespan extension is obtained in flies fed a high carbohydrate/low protein diet, further suggesting that low nutritional protein is a key parameter (Bruce et al., 2013). Similar to findings from yeast, methionine restriction also extends lifespan in Drosophila (Lee et al., 2014). Accordingly, genetic perturbations that decrease the levels of SAM (S-adenosyl methionine), a methionine-derived metabolite, are sufficient to elicit lifespan extension (Obata and Miura, 2015). Of note, in mammalian cells, SAM was shown to signal methionine availability to activate mTORC1 (Gu et al., 2017), hinting for a potentially conserved mechanism in flies. Another important group of AA are branched-chain amino acids (BCAA), consisting of leucine, isoleucine, and valine. BCAA potently induce mTORC1 activity and are hence considered of particular relevance for aging. However, to date, the role of BCAA in aging has been controversial (Juricic et al., 2020). A recent report comparing the effect of BCAA deprivation to simultaneous removal of threonine, histidine and lysine—three EAA that have not been previously associated with lifespan modulation—revealed similar effects between the two AA groups in both TORC1 activity and lifespan extension (Juricic et al., 2020). This study hinted that sufficient TORC1 downregulation upon starvation may be the determining factor for lifespan extension, regardless of which the deprived AA are.

*Studies in rodents:* As observed in *Drosophila*, the nutrient composition of a given diet is more influential than its caloric content for determining the aging and metabolic phenotype also in mouse studies (Solon-Biet et al., 2014). Several studies over the years showed that PR is able to promote lifespan and healthspan extension in mice (Pamplona and Barja, 2006), consolidating PR as a promising anti-aging intervention. Notably, PR modulates mTORC1 activity both in somatic tissues and in tumors, and consequently reduces tumor growth (Lamming et al., 2015). Methionine restriction, in particular, was shown since the 90’s to be sufficient to induce lifespan extension in rats and mice, thus representing an attractive approach that would require restriction of a single EAA (Orentreich et al., 1993; Richie et al., 1994; Zimmerman et al., 2003; Miller et al., 2005; Malloy et al., 2006; Perrone et al., 2013). Accordingly, the DR-induced effect was also blunted by resupplementing the diet with EAA in mice, with the effect being particularly dependent on methionine (Yoshida et al., 2018). Interestingly, circulating or liver methionine levels are substantially lower in the long-lived naked mole rat, as compared to mice, further supporting an important role for methionine in aging (McIsaac et al., 2016). Finally, besides methionine, tryptophan is another EAA that has been linked to lifespan extension (Segall and Timiras, 1976; Segall et al., 1983).

The fact that BCAA are known to influence metabolic health (Fontana et al., 2016; Cummings et al., 2018), implied a similar role in aging. Indeed, lifelong BCAA restriction led to extended lifespan and improved healthspan, specifically in male mice, thus revealing a sex-specific role for BCAA in aging (Richardson et al., 2021). Moreover, transcriptome analyses in skeletal muscles of BCAA-restricted and fully-fed mice revealed downregulated mTOR signaling upon starvation—linked to upregulation of its negative regulators Sestrin2 and Castor1—indicated by reduced phosphorylation of mTORC1 downstream targets (Richardson et al., 2021). Finally, a recent report showed that restriction of dietary isoleucine and valine were of particular importance for metabolic health (Yu et al., 2021), confirming previous data that leucine starvation alone is not able to elicit the beneficial effects of PR (Fontana et al., 2016). Because a low isoleucine diet did not elicit effects in hepatic mTORC1 activity (Yu et al., 2021), whether isoleucine and valine improve metabolic health via distinct mechanisms, or by influencing mTORC1 activity in other tissues, remains to be investigated.

*Studies in humans:* Aging research aims to propose nutritional regimens that improve healthspan, diminish age-related maladies, and—at the same time—can be easily implemented by humans. An important milestone in this endeavor will be the identification of specific macronutrients that are involved in these processes, presumably via modulating mTORC1 activity in metabolically-relevant tissues. In this regard, high nutritional protein consumption is associated with insulin resistance, type 2 diabetes, obesity, increased cancer incidence and mortality in humans (Lagiou et al., 2007; Sluijs et al., 2010; Lagiou et al., 2012; Vergnaud et al., 2013). Interestingly, however, this association is only found for individuals younger than 65 years old (Levine et al., 2014). For individuals over 65, higher protein consumption actually appears to be beneficial and to be associated with reduced cancer risk and mortality, thus pointing to higher protein uptake as a factor required for homeostasis (Levine et al., 2014). Importantly, a small randomized trial showed that a reduction in protein consumption is beneficial for humans by improving metabolic health (Fontana et al., 2016). Future investigations are necessary to conclusively show whether a specific PR or AA-restricted diet can be translated in increased human lifespan and healthspan, and to define the target groups that would benefit the most from such interventions.

## Discussion

Research over the last several decades has consolidated a key role for mTORC1 and the nutrient sensing pathway in aging and in the development of age-related diseases. Accordingly, nutritional, pharmacological or genetic interventions that influence mTORC1 activity, as well as a number of cellular processes that are regulated downstream of mTORC1, are also crucial modulators of lifespan and healthspan. Although more recent work has provided significant insights on how these perturbations mechanistically influence aging, several key questions remain unanswered.

Consistent with the fact that AA availability is a strong activating signal for mTORC1, dietary restriction of certain AA groups—or even of individual AA—is now a well-established anti-aging intervention. However, different AA have been shown to affect mTORC1 in different ways, at variable degrees, and through distinct sensors and signaling cascades (Jewell et al., 2015; Liu and Sabatini, 2020). Comparative studies on the role of various AA in the regulation of mTORC1, and in the modulation of metabolic health over aging are now starting to emerge (Yu et al., 2021), and highlight the need for a more spherical investigation of these crucial cellular processes. At the next level, the functional interplay between AA and other macronutrients that are important for mTORC1 activity, such as sugars and lipids, will also need to be addressed.

Pharmacological or nutritional interventions that are commonly used as anti-aging strategies are known to function via decreasing mTORC1 activity, suggesting that it is the hyperactivation of this complex upon aging that drives the age-related functional decline. Interestingly, a study looking at mTORC1 signaling in tissues of various mouse strains and rats over aging reported tissue-, sex-, and substrate-specific effects, with most tissues showing no obvious increase in mTORC1 signaling upon aging (Baar et al., 2016). Similarly, isoleucine restriction improved metabolic health, without influencing hepatic mTORC1 activity in mice (Yu et al., 2021). Furthermore, in certain tissues, like muscles, mTORC1 activity even declines with age, in part due to the development of anabolic resistance—a major cause for muscle wasting in the elderly—with mTORC1 requiring higher AA amounts to maintain proper cellular metabolic activities (Dardevet et al., 2012). Accordingly, a growing body of evidence shows that increased AA uptake in older individuals may be beneficial for certain aging-related parameters (Levine et al., 2014; Edwards et al., 2015). Further complicating the potentially causal role of mTORC1 in age-related conditions, Rapamycin and DR, two interventions that elicit potent anti-aging effects and are known to limit mTORC1 activity, seem to drive distinct responses, and to control partially non-overlapping processes (summarized in (Unnikrishnan et al., 2020)).

Summarizing, although the nutrient sensing pathways upstream of mTORC1 have been the focus of intense investigation over the last 10–15 years, how AA regulate mTORC1 *in vivo* is poorly understood. By increasing our knowledge on how nutrients signal to regulate mTORC1 in the context of cellular and organismal physiology and aging, we expect that novel, more targeted ways will be discovered that allow us to modulate these key processes in the right organs, at the right time, and to the right extent. Ultimately, this research will come up with suggestions about how our nutritional habits should be modified, and, more importantly, what exactly these pharmacological interventions should be targeting.
